# Neil3 induced neurogenesis protects against prion disease during the clinical phase

**DOI:** 10.1038/srep37844

**Published:** 2016-11-25

**Authors:** Clara M. O. Jalland, Katja Scheffler, Sylvie L. Benestad, Torfinn Moldal, Cecilie Ersdal, Gjermund Gunnes, Rajikala Suganthan, Magnar Bjørås, Michael A. Tranulis

**Affiliations:** 1 Norwegian University of Life Sciences, Faculty of Veterinary Medicine and Biosciences, Campus Adamstuen Oslo, Norway; 2 Department of Cancer Research and Molecular Medicine, Norwegian University of Science and Technology, Trondheim, Norway; 3 Department of Microbiology, Oslo University Hospital and University of Oslo, Norway; 4 Norwegian Veterinary Institute, Oslo, Norway

## Abstract

Base excision repair (BER) is the major pathway for repair of oxidative DNA damage. Mice with genetic knockout of the BER enzyme *Neil3* display compromised neurogenesis in the sub-ventricular zone of the lateral ventricle and sub-granular layer of the dentate gyrus of the hippocampus. To elucidate the impact of oxidative DNA damage-induced neurogenesis on prion disease we applied the experimental prion disease model on Neil3-deficient mice. The incubation period for the disease was similar in both wild type and *Neil3*^−/−^ mice and the overall neuropathology appeared unaffected by Neil3 function. However, disease in the *Neil3*^−/−^ mice was of shorter clinical duration. We observed a mildly reduced astrogliosis in the hippocampus and striatum in the Neil3-deficient mice. Brain expression levels of neuronal progenitor markers, nestin (*Nestin*), sex determining region Box 2 (*Sox2*), Class III beta-tubulin (*Tuj1*) decreased towards end-stage prion disease whereas doublecortin (*Dcx*) levels were less affected. Neuronal nuclei (*NeuN*), a marker for mature neurons declined during prion disease and more pronounced in the *Neil3*^−/−^ group. Microglial activation was prominent and appeared unaffected by loss of Neil3. Our data suggest that neurogenesis induced by Neil3 repair of oxidative DNA damage protects against prion disease during the clinical phase.

Prion diseases such as Creutzfeldt-Jakob disease in humans and scrapie in sheep and goats are invariably fatal, neurodegenerative diseases characterized by protein misfolding and aggregation. The host-encoded cellular prion protein (PrP^C^) misfolds into disease-provoking multimeric aggregates, some of which constitute infectious prions^1^. Proteinase resistant remnants of misfolded prion protein are known as PrP^Sc^ (scrapie). Neuropathological hallmarks of prion disease include tissue vacuolization, deposits of misfolded conformers of PrP, reactive gliosis and neuronal loss^2^,^3^,^4^. Experimental prion diseases in rodents replicate all aspects of naturally occurring prion disease, and are therefore valuable models of protein misfolding associated neurodegeneration. Intracerebral inoculation with the mouse-adapted Rocky Mountain Laboratories (RML) scrapie strain have shown that peak infectivity is reached midway into the asymptomatic incubation period, while glial activation and vacuolization gradually develop, particularly in the thalamus and hippocampus, during the second half of the incubation period^5^. Significant involvement of the hippocampus is a well-known feature of other murine prion models as well^6^ and prion replication has been shown to occur in neural stem cells (NSC) of the sub-granular layer (SGL) of the dentate gyrus of the hippocampus^7^. The SGL is a major site for adult neurogenesis, responding to various insults like ischemia. The role of neurogenesis in protein misfolding associated neurodegeneration, such as Alzheimer’s disease (AD), Parkinson’s disease, Huntington’s disease and prion diseases is not clarified, and data from different model systems sometimes conflict. For instance, both increased^8^ and decreased^9^ hippocampal neurogenesis have been reported in AD. Furthermore, a stage-dependent profile has also been observed, with increased neurogenesis at early stages of neurodegeneration^10^, followed by a decline in stem cell activity at later stages of severe neurodegeneration^11^. The profound neurodegeneration that occurs in the hippocampus of RML-induced prion disease makes this a useful model for studying SGL-driven neurogenesis in a protein misfolding neurodegenerative disease. In a previous study^12^, we examined the development of RML prion disease in mice with combined knockout of DNA repair enzymes, Mutyh and Ogg1, which initiate base excision repair (BER) of reactive oxygen species (ROS)-mediated DNA damage^13^. The BER pathway is initiated by DNA glycosylases recognizing modified bases^14^, including the mammalian NEIL family, where the three members (Neil 1, 2, 3)^13^,^15^,^16^ are homologous to the *E. coli* formamidopyrimidine DNA glycosylase and endonuclease VIII (Nei) enzymes^17^,^18^. The full spectrum of physiological activities of the Neil enzymes has not been clarified and recent data suggest roles beyond DNA repair and genomic stability, possibly related to gene regulatory activities^19^,^20^. *In vitro* Neil1 and Neil2 display repair activity on both double-stranded DNA and single-stranded DNA (ssDNA) substrates. Neil3 exhibits DNA glycosylase activity and AP-lyase activity specific for ssDNA^21^ and is considered the main DNA glycosylase for removal of hydantoins in ssDNA^22^. The phenotype of mice that are deficient in Neil3 is associated with impaired proliferative capacity of the neural progenitor cells^19^,^20^. In mouse, *Neil3* is highly expressed in neural stem cells, such as SGL and the sub-ventricular zone^23^,^24^,^25^. Interestingly, *Neil3*^−/−^ mice have a reduced capacity for hippocampal neurogenesis after hypoxia-induced neuropathology^19^. Indeed, Neil3-dependent DNA repair appears essential for maintenance of neural stem cell proliferative capacity, which indicates that repair of oxidative DNA damage in NSCs is required for adult neurogenesis^20^. Surprisingly, Neil3 deficient mice showed no change in steady state levels of oxidative DNA damage and genome integrity, indicating a role beyond canonical BER. It thus, appears that Neil3 deficient mice are an interesting model for studying the impact of impaired neurogenesis in neurodegenerative disease. Therefore, in order to broaden our understanding of hippocampal neurogenesis during neurodegeneration, we report a study of prion disease in mice with genetic knockout (KO) of *Neil3*.

## Results

**Figure 1:**
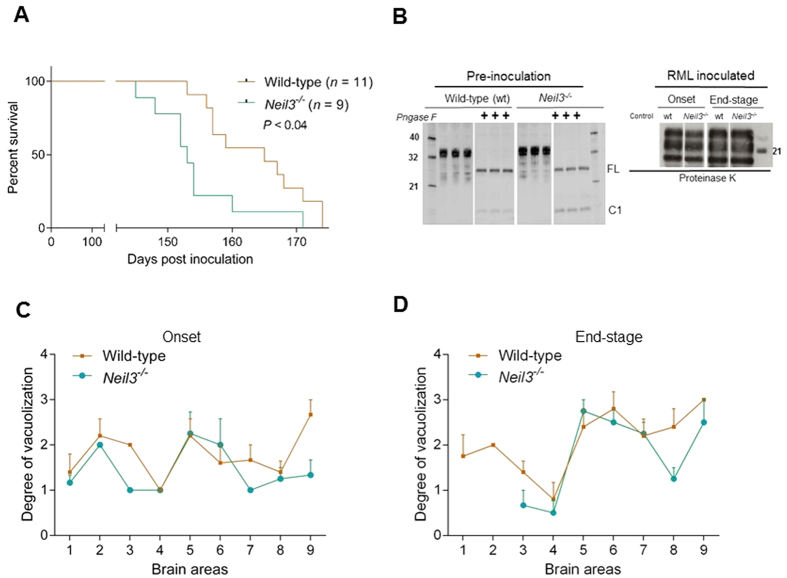
(**A**) Survival plot of wild-type (*n* = 11) and *Neil3*^−/−^ (*n* = 9) mice after intracerebral inoculation with the Rocky Mountain Laboratories (RML) strain of prions. The preclinical phase lasted for around 138 days in both groups of animals, but the *Neil3*^−/−^ mice suffered a shorter clinical phase of disease (*p* < 0.05, Log rank Mantel Cox test). (**B**) Western immune blots of whole brain PrP pre inoculation (left panel, mAb Bar224) and proteinase resistant PrP^Sc^ at onset and end-stage (right panel, mAb SHa31). The steady-state levels of PrP at pre-inoculation was similar between the groups and dominated by di-glycosylated PrP. Levels of full length (FL) the C-terminal truncation product (C1) was also similar between the groups. The banding pattern of PrP^Sc^ as seen after PK treatment was also similar between the groups at both onset and end-stage of disease. (**C**,**D**) Lesion profile. Distribution of brain vacuolization in nine defined areas (1 medulla oblongata, 2 cerebellar cortex, 3 cortex of the superior colliculus, 4 hypothalamus, 5 thalamus, 6 hippocampus, 7 septal nuclei, 8 cerebral cortex dorsal to corpus callosum, 9 cerebral cortex dorsal to septal nuclei) of the brain as observed at onset (**C**) and at end-stage (**E**) in wild-type (*n* = 5) and Neil3 (*n* = 4–6) KO mice. Vacuolization scores: 0 no vacuoles, 1 few vacuoles unevenly distributed, 2 few to moderate number of vacuoles evenly distributed, 4 many vacuoles with some confluence, 5 dense and confluent vacuolization.

All RML-inoculated mice developed neurological signs, and prion disease was confirmed by histopathological analysis. The first signs of clinical disease, kyphosis, pelvic limb weakness and lethargy, appeared around 138 days post inoculation (dpi) in both groups. We observed that *Neil3*^−/−^ mice had a shorter clinical phase than the wild-type mice, with mean survival times from onset to end-stage being 18 and 22 days for *Neil3*^−/−^ and wild-type mice respectively (*p* = 0.04). Kaplan Meier survival plots also revealed statistically different survival curves (Fig. 1A). One of the *Neil3*^−/−^ mice, however, had a more prolonged clinical course, similar to that of the wild-type mice, in which more than 50% of the animals were still alive 18 days after onset of disease.

Analysis of steady state levels of PrP^C^ in brain by western immune blots revealed similar levels of predominantly di-glycosylated PrP^C^ and a similar level of proteolytic processing, as judged by full length (FL) and C-terminal fragment 1 (C1) between the groups (Fig. 1B, left panel). Proteinase K resistant PrP banding patterns (PrP^Sc^ type) were similar at onset and end-stage in both groups (Fig. 1B, right panel).

Distribution of vacuolization in 9 brain areas at onset (Fig. 1C) and end-stage (Fig. 1D) were most prominent in the cerebral cortex, the thalamus and hippocampus as expected for the RML prion strain and in agreement with^26^,^12^.

**Figure 2:**
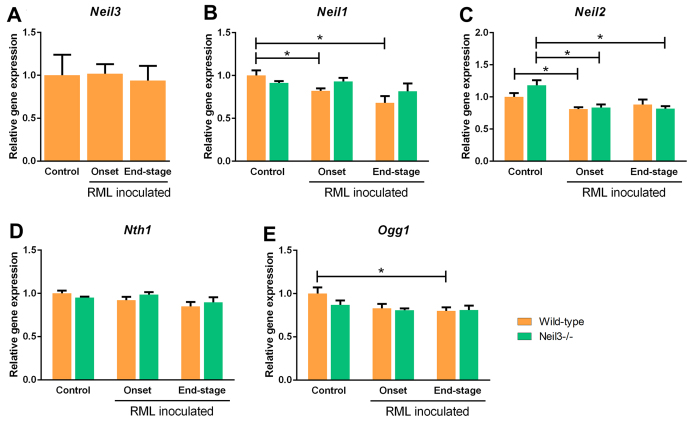
Expression of DNA glycosylases: nei endonuclease VIII-like, *Neil1*, *Neil2*, *Neil3*, DNA endonuclease III-like, *Nth1* and 8-oxoguanine DNA glycosylase, *Ogg1* in brain at onset and end-stage prion disease (*n *= 4–6, both groups), relative to expression in un-inoculated age-matched controls of wild-type mice (*n *= 4–6). Figure shows mean values with SEM.

Expression of DNA glycosylases, *Neil1*, *Neil2*, *Neil3*, *Nth1* and *Ogg1,* in brain at onset and at end-stage prion disease, relative to expression in un-inoculated age-matched controls of wild-type mice, is given in Fig. 2. There was a significant decrease in expression at either onset or end-stage or both for some of the DNA glycosylases (Neil1, Neil2 and Ogg1) in wild-type mice, demonstrating a lack of compensatory up-regulation of brain DNA glycosylases in prion disease.

**Figure 3:**
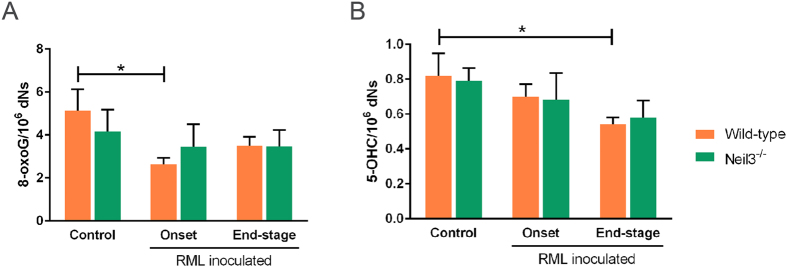
Mass spectrometry measurement of genomic 8-oxo guanine (8-oxoG) and 5-hydroxy cytosine (5-OHC) levels in whole brain of un-inoculated controls of wild-type (*n *= 4–6) and Neil3 KO (*n *= 4–6) mice and RML inoculated mice of both groups at onset (*n *= 4–6, both groups) and at end-stage (*n *= 4–6, both groups). Figure shows mean values with SEM.

Accumulation of the oxidative DNA base lesions 8-oxoG and 5-OHC at onset and end-stage prion disease was measured by mass spectrometry. The results, presented in Fig. 3, show no global increase of these lesions during prion disease or differences between the genotypes, indicating that Neil3 is not affecting the steady state level of 8-oxoG and 5-OHC, in which 5-OHC is substrate for Neil3. It thus appears that the Neil3 DNA glycosylase is not important for removing oxidative DNA base lesions genome-wide during prion disease.

**Figure 4:**
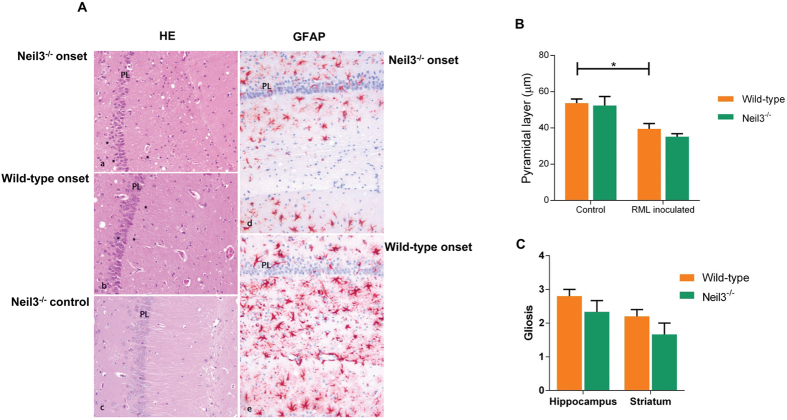
Histopathology (hematoxylin eosin) and astrogliosis (glial fibrillary acidic protein, GFAP) in hippocampus at onset of disease (**A**) in Neil3 KO and in wild-type mice inoculated with RML and in un-inoculated Neil3 KO control mice. The level of vacuolization is moderate and evenly distributed in both inoculated groups. Magnification: 200X. There was a reduction of the thickness of the pyramidal cell layer at onset of disease (**B**) in both groups, (*n* = 4 in each group). The astrocyte proliferation appeared more prominent in wild-type mice as compared to Neil3 KO mice in the hippocampus and in the striatum (**A**,**C**) (*n* = 4, both groups). Figure shows mean values with SEM (**B**,**C**).

Vacuolization was present in both groups and progressed similarly through the clinical phases. There was loss of pyramidal neurons already at onset of disease in both wild-type and *Neil3*^−/−^ mice (Figs 1C and 4B) leading to a reduced thickness of the layer compared with controls. This neuronal loss was accompanied by vacuolization and astrogliosis (Fig. 4A, right panels), covering all layers of the hippocampus. The astrogliosis appeared more prominent in the wild-type mice than the *Neil3*^−/−^ mice in both hippocampus and striatum (Fig. 4C).

**Figure 5:**
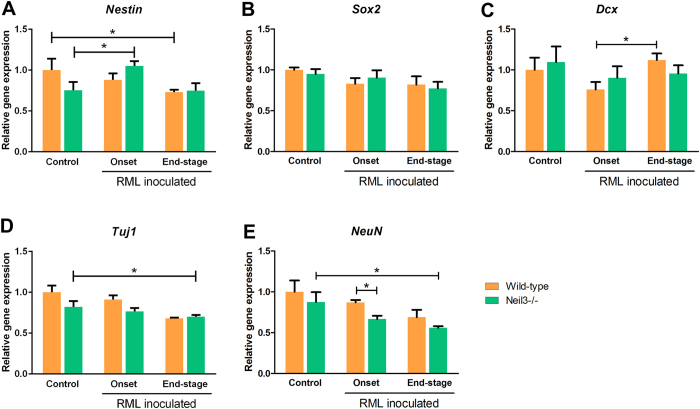
Whole brain expression levels of neuronal stem cell marker Nestin (*Nes*), Sex determining region Y box (*Sox2*) and immature neuron marker Doublecortin (*Dcx*), Class III beta-tubulin (*Tuj1*) and mature neuron marker Feminizing locus on X-3 (*NeuN*) in wild-type and Neil3 KO mice at onset and end-stage of prion disease, relative to age-matched un-inoculated controls. In both groups, as expected a significant drop in mature neuron marker *NeuN* was observed already at onset of disease and more prominent at end-stage. Expression levels of *Dcx* were mildly up-regulated at end-stage as compared to onset in both groups. *N* = 4–6 in all groups, data presented as means with SEM.

**Figure 6:**
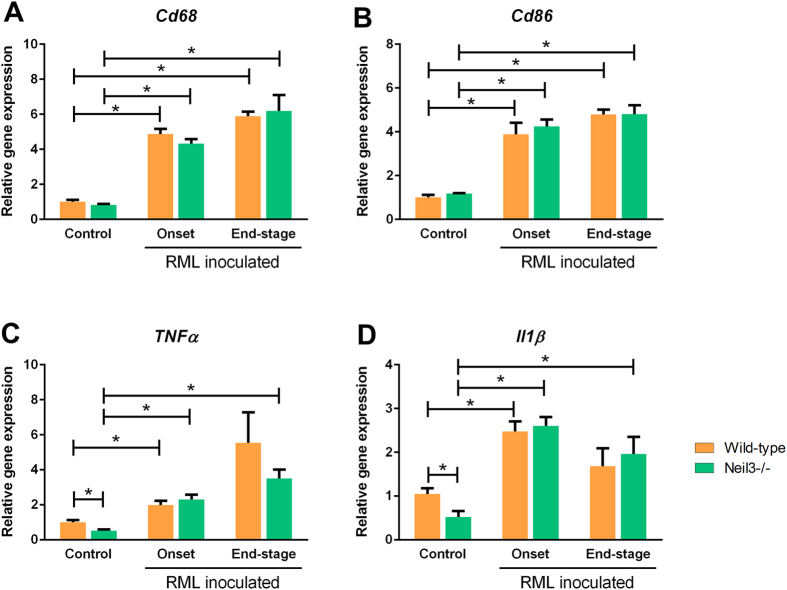
Whole brain expression levels of microglial gene markers, at onset and end-stage prion disease, relative to un-inoculated controls: Cluster of differentiation 68 (CD68) and 86 (CD86), tumor necrosis-factor alpha (TNF*α*) and interleukin 1-beta (Il1*ß*). N = 4–6 in all groups. There is a profound increase in all markers at onset of disease, which appears similar between controls and Neil3 KO mice. With the exception of Il1ß, which decreases slightly towards end-stage, all markers increase further towards end-stage prion disease.

The expression of neuronal progenitor markers Nestin (*Nestin*), Sex determining region Y box (*Sox2*) and Class III beta-tubulin (*Tuj1*), were all maintained at normal levels at onset of disease, however, declining towards end-stage (Fig. 5A,B,D). Doublecortin (*Dcx*), a neuroblast and immature neuron marker, was significantly up-regulated at end-stage compared with onset in wild-type mice (Fig. 5C), while the mature neuron marker, Neuronal Nuclei (*NeuN*), was decreased in wild-type and Neil3 KO mice at onset and end-stage of prion disease, due to severe neuronal loss (Fig. 5D). At onset, expression of *NeuN* was significantly lower in *Neil3* KO mice compared with wild-type. Microglial activation was assessed by expression levels of Clusters of differentiation 68 and 86 (*Cd68* and *Cd86*), tumor necrosis-factor alpha (*TNFα*) and interleukin 1-beta (*Il1ß*) (Fig. 6A–D). All genes were markedly upregulated at onset of disease and continued to rise towards end-stage prion disease, except *Il1ß* that declined slightly towards end-stage.

## Discussion

The mouse Neil3 DNA glycosylase is required for maintenance and differentiation of neuronal stem progenitor cells during ageing and in response to acute hypoxia/ROS-mediated stress^19^,^20^. Studies in rat demonstrated that genetic knockdown of the Ogg1 and Neil3 DNA glycosylases mitigates pluripotency and initiates premature senescence of multipotent neural progenitor cells^27^. In this study, we demonstrate that Neil3 protects the brain during the clinical phase of prion disease. The full impact of neurogenesis as a counteracting homeostatic mechanism in slowly developing neurodegenerative conditions, such as AD and prion diseases, has not been clarified. Since experimental prion diseases in rodents are considered *bone fide* prion diseases, reproducibly recapitulating all major aspects of naturally occurring prion disease, we wanted to compare overall disease development and characteristics in normal and neurogenesis-compromised mice, like the Neil3 KO line. The involvement of hippocampal SGL early in the pre-clinical disease development and the subsequent severity of neuronal death in this brain area in RML prion disease renders this a particularly well-suited disease model for studying SGL neurogenesis in the face of prion-driven neuronal death.

We have previously studied RML prion disease in mice severely deficient in DNA repair, by combined knockout of Ogg1 and Mutyh and observed that several disease features appeared unaffected by the DNA repair deficiency^12^. However, the clinical phase of the disease was more severe and shorter in the repair-compromised mice, suggesting that DNA integrity is challenged in the clinical, toxic phase of prion disease. Interestingly, data presented here show that the Neil3 deficient mice share several similarities with the Ogg1 and Mutyh double knockouts, with a shorter and more dramatic clinical disease, but without other major alterations in disease characteristics, such as distribution of brain lesions. Despite the fact that DNA repair capacities appears to be gradually overwhelmed during prion disease, neither this nor the lack of Neil3 elicit any compensatory increase in expression levels of other DNA glycosylases with overlapping substrate specificities (i.e. Neil1 and Neil2).

It has been shown that during prion disease, neuronal stem cells are heavily affected and accumulate PrP^Sc^ and could thus potentially contribute to disease progression^7^. Importantly, it was also shown that prion disease skewed the neuronal fate of neuronal progenitors, with loss of neuroblasts and immature neurons and an increase in astrocytes. Thus, in prion disease, adult capacities for neurogenesis are distorted and overrun by neuronal loss. This effect could even out differences in neurogenesis between wild-type and Neil3-deficient mice, at least during the toxic/clinical phase of prion disease. However, our observations of similar time of onset of disease suggest that neurogenesis probably has a minor effect in halting disease progression also in the pre-clinical phase. Gomez-Nicola *et al*. suggest a biphasic model of neurogenesis, with a significant increase in the number of proliferative cells in the subgranular layer, which increases as the disease progresses^10^. At the terminal stage, the neurogenic process is fading, as the Dcx+ cells in dentate gyrus are not observed.

Interestingly, we observed a mildly reduced astrogliosis in the Neil3 KO mice compared with the wild-type controls. This might reflect reduced proliferative capacity in the Neil3-deficient mice. Analysis of neuronal progenitor markers *Nestin*, *Sox2*, *Tuj1* and neuroblast marker *Dcx* revealed little difference between wildtype and Neil3 KO mice. All progenitor markers declined towards end-stage prion disease, except *Dcx* that was up-regulated in wild-type mice at end-stage, compared with onset of disease. This upregulation of Dcx was not evident in Neil3 deficient mice, supporting a role of Neil3 in activating neurogenesis during the clinical phase in response to neuronal loss. Expression levels of *NeuN*, a marker for mature neurons, dropped significantly more in Neil3-deficient brain than wild type during prion disease, probably due to severe neuronal loss, indicating that the neurogenic capacity is impaired in Neil3 KO. However, detailed morphometric and gene expression analysis using laser micro-dissected tissue samples or by *in situ* hybridization techniques are needed for comprehensive assessment of neurogenesis at different stages of prion derived brain pathology in Neil 3 KO and in wild-type mice.

It has recently been demonstrated that activation of microglia contributes an over-all protective role against prion pathology, probably by phagocytosing harmful prion aggregates or cellular debris^28^. Our observation of shortened clinical duration in Neil3 KO mice could therefore be a result of compromised microglial activation in this genotype. In order to evaluate the activation state of microglia at onset and end-stage prion disease in wild-type and Neil3 Ko mice we compared expression levels of microglial markers *Cd68*, *Cd86* and inflammation markers *TNFα* and *Il1ß* at both time-points. As expected, already at onset of disease a marked microglial activation in both genotypes that increases even further towards the final stages of the disease is evident. Our data do not demonstrate major differences in microglial activation between wildtype and Neil3 KO mice, suggesting that loss of Neil3 does not compromise microglial activation and proliferation during prion-induced neurodegeneration.

Our observations of a significantly shortened clinical phase of prion disease in mice compromised in different BER enzyme activities, such as *Mutyh* and *Ogg1*^12^ and here in *Neil3* deficient mice suggest that during clinical prion disease, DNA glycosylase activities provide neuroprotection. Since the physiological roles of these enzymes are diverse and partly unknown it is of considerable interest to elucidate underlying pathogenic commonalities that might reveal in molecular detail how DNA glycosylases prolong neuronal survival during the toxic phase of prion disease. The precise mechanisms by which DNA glycosylases contributes neuroprotection during prion disease awaits a deeper understanding of the physiological roles of these enzymes in the brain, beyond maintenance of genomic stability or canonical repair.

## Materials and Methods

### Ethics statement

This study was carried out in accordance with the Norwegian Regulation on Animal Experimentation, which is based upon the European Convention for the Protection of Vertebrate Animals Used for Experimental and Other Scientific Purposes, and approved by the Norwegian Animal Research Authority, approval number 6232.

### Mice

In these experiments, 36 female mice, 20 C57 Bl/6Y (wild-type) and 16 transgenic *Neil3*^−/−^ mice, were inoculated intra-cerebrally with the RML isolate. The mice weighed between 18 and 22 grams at inoculation. Four mice were excluded from the experiment. Three mice, two wild-type and one *Neil3*^−/−^, died within two days after inoculation due to complications and one was found dead 156 dpi, but material was of insufficient quality for further diagnosis and analysis. Four wild-type mice and three *Neil 3*^−/−^ mice served as non-inoculated age-matched controls for the DNA-damage analysis. Wild-type C57 Bl/6 mice were bred in-house in the same room as *Neil 3*^−/−^ mice. Mice were housed in groups of four in individually ventilated and sealed cages, under a 12-h light/ 12-h dark cycle at 21 °C, with food and water ad libitum. Animals were bred in accordance with European regulations FELASA category C. The genotypes of the mice were routinely tested by a PCR-based protocol.

### Inoculum

In this study, mouse adapted scrapie strain RML, which is originally derived from sheep scrapie brain homogenate (SSBP-1) was provided by Dr Andreoletti (ENV Toulouse, France). This prion strain has been passaged in many lines of mice for many decades and adapted into C57Bl/6Y. The mice were anesthetized with a mixture of tiletamine, zolezepam and xylazine that was injected subcutaneously, and 20 μl of a 10% inoculum was injected into the right midhemisphere.

### Monitoring and sampling

The mice were monitored daily and signs of clinical disease, such as stiff tail, ataxia, hyperexcitability, ruffled coat, kyphosis, paraparesis, trembling and lethargy, were recorded.

In order to characterize the progression of disease parameters from onset to end-stage prion disease, 8 C57Bl/6Y (wild-type) and 8 transgenic *Neil3*^−/−^ mice were euthanized at onset of early clinical signs. When the predefined clinical endpoint was reached, 9 C57Bl/6Y (wild-type) and 7 transgenic *Neil3*^−/−^ mice were killed by dislocation of the craniocervical junction and sampled. The brain was cut longitudinally. One half of the brain was fixed in 10% neutral buffered formalin. After fixation, the brain tissue was cut into five transverse sections as described by Bruce and co-workers^29^ and processed for histopathological analysis and immunohistochemical (IHC) detection of PrP^Sc^ and the astrocyte marker, glial fibrillary acidic protein (GFAP). The second half of the brain was frozen and later used for detection of PrP^Sc^ by western blot (WB).

For the DNA damage analysis, one-half of the brain and the spleen were sampled into RNAlater, to stabilize and protect cellular RNA. The second half was used for histo-pathological and IHC analysis.

### Lesion scoring and immunohistochemistry

After fixation and processing, the brain tissue was cut and embedded in paraffin. Sections (4 μm thickness) were routinely deparaffinized, rehydrated and stained with hematoxylin and eosin (HE). The following brain areas were blindly scored for lesion profiling by two pathologists; dorsal half of the medulla oblongata, the cerebellar cortex of the folia, the cortex of the superior colliculus, hypothalamus, thalamus, hippocampus, the septal nuclei, cerebral cortex, dorsal to the corpus callosum and the cerebral cortex dorsal to the septal nuclei. The degree of vacuolization was scored from zero (no vacuoles) to five (dense vacuolization). The thickness of the hippocampal pyramidal layer was measured blindly using LAS camera software at six different points in each section and the mean was calculated. The intensity of GFAP labelling was semi-quantitatively scored according to the following criteria: 0, negative; 1, little; 2, moderate; 3, strong labelling.

For detection of PrP^Sc^, the sections were pretreated by immersion in 98% formic acid for 30 min, washed and autoclaved at 121 °C in 0.01 M citric acid, pH 6.1 for 30 min, prior to incubation with proteinase K (4 μg/ ml) at 37 °C for 5 minutes. A cocktail of primary antibodies (F89/160.1.5 1/2000 and 2G11 1:400) was applied for 1 h at room temperature and for visualization of PrP^Sc^, a commercially available kit (EnVision^®^+ System–HRP (AEC) Mouse, DAKO) was used. Staining with GFAP was performed with a polyclonal rabbit anti-cow GFAP antibody (Dakopatts) at 1: 2000 dilution for 1 h at room temperature and detection by use of the kit EnVision^®^+ System-HRP (AEC) Rabbit (DAKO).

### Western blot analysis for detection of PrP^Sc^

Brain samples were homogenized at room temperature in a buffer containing Tris-HCl pH 7.4, 150 mM NaCl, 0.5% (w/v) Triton X-100, 0.5% sodium deoxycholate, and treated with Proteinase K (4 μg/ml) for 1 h at 37 °C, or with PNGase F (New England Biolabs) for 2 h at 37 °C for deglycosylation. Samples were boiled in SDS sample buffer and run on 12% pre-cast polyacrylamide gels (Bio-Rad) and transferred to polyvinylidene fluoride membranes by a semi-dry blotter. Membranes were blocked for 1 h in 5% fat-free dried milk, and incubated with primary antibody Bar224 (Spi Bio, France) overnight at 4 °C. Secondary antibody (goat anti-mouse) labeled with alkaline phosphatase (ALP: GE Healthcare, UK) was used to visualize bands with a fluorescence imager (Typhoon 9200, GE Healthcare, UK) after incubation with the ALP substrate (ECFTM, Western blotting reagent pack, GE Healthcare). In addition, an EU and OIE approved test, (TeSeE WESTERN BLOT, Bio-Rad) was used according to the recommendations of the manufacturers. The immunodetection was performed using the monoclonal antibody SHa31 that recognizes the amino acids (YEDRYYRE), corresponding to codons 145–152 in human PrP. Peroxidase activity was revealed using enhanced chemiluminescence (ECL, GE Healthcare, UK) substrate.

### Transcription analysis of DNA glycosylases

Brain hemispheres were stored in RNAlater for at least 24 h before homogenization with FastPrep-24 (MP Biomedical). Total RNA was isolated using the Allprep DNA/RNA/Protein isolation kit (Qiagen) and 1 μg reversely transcribed into cDNA with High Capacity cDNA Reverse Transcription Kit (Applied Biosystems). Gene targets and corresponding primers for quantitative amplifications are given in Table 1. Relative expression levels were calculated using the comparative ∆ct method and related to the housekeeping gene *ß-actin*.

### LC-MS/MS quantification of 8-oxodG and 5ohdC

DNA samples were digested by incubation with a mixture of nuclease P1 from *Penicillium citrinum* (Sigma, N8630), DNaseI (Roche, 04716728001) and ALP from *E. coli* (Sigma P5931) in 10 mM ammonium acetate buffer pH 5.3, 5 mM MgCl_2_ and 1 mM CaCl_2_ for 30 min at 40 °C. The samples were methanol precipitated, supernatants were vacuum centrifuged at room temperature until dry, and dissolved in 50 μl of water for LC/MS/MS analysis. Quantification was performed with the use of an LC-20AD HPLC system (Shimadzu) coupled to an API 5000 triple quadrupole (ABSciex) operating in positive electrospray ionization mode. The chromatographic separation was performed with the use of an Ascentis Express C18 2.7 μm 150 × 2.1 mm i.d. column protected with an Ascentis Express Cartridge Guard Column (Supelco Analytical) with an Exp Titanium Hybrid Ferrule (Optimize Technologies Inc.). The mobile phase consisted of A (water, 0.1% formic acid) and B (methanol, 0.1% formic acid) solutions. The following conditions were employed for chromatography: for unmodified nucleosides – 0.13 mL/min flow, starting at 10% B for 0.1 min, ramping to 60% B over 2.4 min and re-equilibrating with 10% B for 4.5 min; for 5-oh(dC) - 0.14 mL/min flow, starting at 5% B for 0.1 min, ramping to 70% B over 2.7 min and re-equilibrating with 5% B for 5.2 min; for dC modifications, and 8-oxo(dG) - 0.14 mL/min flow, starting at 5% B for 0.5 min, ramping to 45% B over 8 min and re-equilibrating with 5% B for 5.5 min. For mass spectrometry detection the multiple reaction monitoring (MRM) was implemented using the following mass transitions: 252.2/136.1 (dA), 228.2/112.1 (dC), 268.2/152.1 (dG), 243.2/127.0 (dT), 244.1/128 [5-oh(dC)], 284.1/168.1 [8-oxo(dG)].

### Statistical analysis

Graphical presentations and statistical analyses were performed with the GraphPad Prism 6 software package. *P*-values < 0.05 were considered statistically significant. Kaplan-Meier survival plots were compared with Log Rank Mantel Cox test, whereas mean values of DNA damage, morphometric data and gene expression comparisons were analyzed by Student’s *t*-test.

## Additional Information

**How to cite this article**: Jalland, C. M. O. *et al*. Neil3 induced neurogenesis protects against prion disease during the clinical phase. *Sci. Rep.* **6**, 37844; doi: 10.1038/srep37844 (2016).

**Publisher's note:** Springer Nature remains neutral with regard to jurisdictional claims in published maps and institutional affiliations.

## Figures and Tables

**Table 1:** Gene targets and corresponding primer sets used for quantitative amplification.

Target	Forward	Reverse
*Neil1*	GCTGACCCTGAGCCAGAAGAT	CCCCAACTGGACCACTTCCT
*Neil2*	TTTAGTGGTGGTGGCTTCCT	TGATGTTCCCTAATCCTGAGAAG
*Neil3*	GGGCAACATCATCAAAAATGAA	CTGTTTGTCTGATAGTTGACACACCTT
*Nth1*	CCCGGAGCCGTTGCA	TGCTCTCCAGCCAGACCAA
*Ogg1*	GTGACTACGGCTGGCATCC	AGGCTTGGTTGGCGAAGG
*Nestin*	TCTCCAGAAGAGGAGGACCA	TTCGAGAGATTCGAGGGAGA
*Sox2*	TCCAAAAACTAATCACAACAATCG	GAAGTGCAATTGGGATGAAAA
*Dcx*	TACCTGGGATTTTCCTTTGG	CTCGTTCGTCAAAATGTCCA
*Tuj-1*	CCAAGACAAGCAGCATCTGT	CAGAGCCAAGTGGACTCACA
*Rbfox3* (*NeuN*)	GAGTCTATGCCGCTGCTGAT	TTGCTAGTAGGGGGTGAAGC
*Cd68*	CCAATTCAGGGTGGAAGAAA	ATGGGTACCGTCACAACCTC
*Cd86*	CTCTTTCATTCCCGGATGGT	GGAGGGCCACAGTAACTGAA
*Tnfα*	ACGGCATGGATCTCAAAGAC	GTGGGTGAGGAGCACGTAGT
*Il1β*	CAGGCAGGCAGTATCACTCA	TGTCCTCATCCTGGAAGGTC

## References

[b1] Prusiner S. B. Molecular biology and pathogenesis of prion diseases. Trends in Biochemical Sciences 21, 482–487, doi: 10.1016/S0968-0004(96)10063-3 (1996). 9009832

[b2] Masters c. L. & Richardson E. P. Subacute Spongiform Encephalopathy (Creutzfeldt-Jakob Disease). The Nature And Progression Of Spongiform Change 101, 333–344, doi: 10.1093/brain/101.2.333 (1978). 352478

[b3] Williams A., Lucassen P. J., Ritchie D. & Bruce M. PrP Deposition, Microglial Activation, and Neuronal Apoptosis in Murine Scrapie. Experimental Neurology 144, 433–438, doi: 10.1006/exnr.1997.6424 (1997). 9168844

[b4] Soto C. & Estrada L. D. PRotein misfolding and neurodegeneration. Archives of Neurology 65, 184–189, doi: 10.1001/archneurol.2007.56 (2008). 18268186

[b5] Sandberg M. K., Al-Doujaily H., Sharps B., Clarke A. R. & Collinge J. Prion propagation and toxicity *in vivo* occur in two distinct mechanistic phases. Nature 470, 540–542 (2011). 21350487 10.1038/nature09768

[b6] Jeffrey M. . Synapse loss associated with abnormal PrP precedes neuronal degeneration in the scrapie-infected murine hippocampus. Neuropathology and Applied Neurobiology 26, 41–54, doi: 10.1046/j.1365-2990.2000.00216.x (2000). 10736066

[b7] Relaño-Ginès A. . Prion replication occurs in endogenous adult neural stem cells and alters their neuronal fate: involvement of endogenous neural stem cells in prion diseases. PLoS Pathog 9, e1003485 (2013). 23935493 10.1371/journal.ppat.1003485 PMC3731238

[b8] Jin K. . Increased hippocampal neurogenesis in Alzheimer’s disease. Proceedings of the National Academy of Sciences 101, 343–347 (2004). 10.1073/pnas.2634794100 PMC314187 14660786

[b9] Markiewicz I., Sypecka J., Domanska-Janik K., Wyszomirski T. & Lukomska B. Cellular Environment Directs Differentiation of Human Umbilical Cord Blood–Derived Neural Stem Cells *In Vitro*. Journal of Histochemistry & Cytochemistry 59, 289–301 (2011). 21378283 10.1369/0022155410397997 PMC3201151

[b10] Gomez-Nicola D. . Temporal dynamics of hippocampal neurogenesis in chronic neurodegeneration. Brain 137, 2312–2328 (2014). 24941947 10.1093/brain/awu155 PMC4107745

[b11] Chen Q. . Adult neurogenesis is functionally associated with AD-like neurodegeneration. Neurobiology of disease 29, 316–326 (2008). 17980611 10.1016/j.nbd.2007.09.005 PMC2254142

[b12] Jalland C. M. O. . Accelerated clinical course of prion disease in mice compromised in repair of oxidative DNA damage. Free Radical Biology and Medicine 68, 1–7, doi: 10.1016/j.freeradbiomed.2013.11.013 (2014). 24296244

[b13] Barnes D. E. & Lindahl T. Repair And Genetic Consequences Of Endogenous Dna Base Damage In Mammalian Cells. Annual Review of Genetics 38, 445–476 (2004). 10.1146/annurev.genet.38.072902.092448 15568983

[b14] Krokan H. E. & Bjørås M. Base excision repair. Cold Spring Harbor perspectives in biology 5, a012583 (2013). 23545420 10.1101/cshperspect.a012583 PMC3683898

[b15] Bandaru V., Sunkara S., Wallace S. S. & Bond J. P. A novel human DNA glycosylase that removes oxidative DNA damage and is homologous to Escherichia coli endonuclease VIII. DNA Repair 1, 517–529, doi: 10.1016/S1568-7864(02)00036-8 (2002). 12509226

[b16] Hazra T. K. . Identification and characterization of a human DNA glycosylase for repair of modified bases in oxidatively damaged DNA. Proceedings of the National Academy of Sciences 99, 3523–3528, doi: 10.1073/pnas.062053799 (2002). PMC122556 11904416

[b17] Wallace S. S., Bandaru V., Kathe S. D. & Bond J. P. The enigma of endonuclease VIII. DNA Repair 2, 441–453, doi: 10.1016/S1568-7864(02)00182-9 (2003). 12713806

[b18] Zharkov D. O., Shoham G. & Grollman A. P. Structural characterization of the Fpg family of DNA glycosylases. DNA Repair 2, 839–862, doi: 10.1016/S1568-7864(03)00084-3 (2003). 12893082

[b19] Sejersted Y. . Endonuclease VIII-like 3 (Neil3) DNA glycosylase promotes neurogenesis induced by hypoxia-ischemia. Proceedings of the National Academy of Sciences of the United States of America 108, 18802–18807, doi: 10.2307/23058555 (2011). 22065741 PMC3219145

[b20] Regnell Christine, E. . Hippocampal Adult Neurogenesis Is Maintained by Neil3-Dependent Repair of Oxidative DNA Lesions in Neural Progenitor Cells. Cell Reports 2, 503–510, doi: 10.1016/j.celrep.2012.08.008 (2012). 22959434

[b21] Takao M. . Human Nei-like protein NEIL3 has AP lyase activity specific for single-stranded DNA and confers oxidative stress resistance in Escherichia coli mutant. Genes to Cells 14, 261–270, doi: 10.1111/j.1365-2443.2008.01271.x (2009). 19170771

[b22] Kranich J. . Engulfment of cerebral apoptotic bodies controls the course of prion disease in a mouse strain–dependent manner. The Journal of Experimental Medicine 207, 2271–2281, doi: 10.1084/jem.20092401 (2010). 20837697 PMC2947076

[b23] Torisu K., Tsuchimoto D., Ohnishi Y. & Nakabeppu Y. Hematopoietic Tissue–Specific Expression of Mouse Neil3 for Endonuclease VIII–Like Protein. Journal of Biochemistry 138, 763–772, doi: 10.1093/jb/mvi168 (2005). 16428305

[b24] Rolseth V. . Widespread distribution of DNA glycosylases removing oxidative DNA lesions in human and rodent brains. DNA Repair 7, 1578–1588, doi: 10.1016/j.dnarep.2008.06.007 (2008). 18603019 PMC3842036

[b25] Hildrestrand G. . Expression patterns of Neil3 during embryonic brain development and neoplasia. BMC Neurosci 10, 1–8, doi: 10.1186/1471-2202-10-45 (2009). 19426544 PMC2686684

[b26] Fraser H. & Dickinson A. G. The sequential development of the brain lesions of scrapie in three strains of mice. Journal of Comparative Pathology 78, 301–311, doi: 10.1016/0021-9975(68)90006-6 (1968). 4970192

[b27] Reis A. & Hermanson O. The DNA glycosylases OGG1 and NEIL3 influence differentiation potential, proliferation, and senescence-associated signs in neural stem cells. Biochemical and Biophysical Research Communications 423, 621–626, doi: 10.1016/j.bbrc.2012.04.125 (2012). 22564741

[b28] Zhu C. . A neuroprotective role for microglia in prion diseases. The Journal of experimental medicine 213, 1047–1059 (2016). 27185853 10.1084/jem.20151000 PMC4886355

[b29] Bruce M. & Dickinson A. Dementia and unconventional slow infections. Psychopharmacology of old age 15–23 (1982).

